# Machine learning for predicting climate change impacts on *Pseudopithomyces chartarum* spore counts: a risk indicator of facial eczema

**DOI:** 10.1080/00480169.2025.2579134

**Published:** 2025-11-09

**Authors:** M Wada, O Sagarasaeranee, N Cogger, J Marshall, E Cuttance, G Macara, A Sood, E Vallee

**Affiliations:** a EpiCentre, Tāwharau Ora – School of Veterinary Science, Massey University, Palmerston North, New Zealand; b Department of Livestock Development, Ministry of Agriculture, Bangkok, Thailand; c School of Mathematical and Computational Sciences, Massey University, Palmerston North, New Zealand; d VetEnt, Te Awamutu, New Zealand; e EpiVets Ltd, Te Awamutu, New Zealand; f National Institute of Water and Atmospheric Research, Wellington, New Zealand

**Keywords:** Facial eczema, environmental health, livestock disease, climate change, sustainable agriculture, disease forecasting, fungal spores

## Abstract

**Aims:**

To determine the importance of 11 climate variables on pasture spore count of *Pseudopithomyces chartarum*, a risk indicator of facial eczema (FE), and to forecast spore counts in New Zealand until 2100, using longitudinal *P. chartarum* pasture spore count data.

**Methods:**

Between 2010 and 2017, spore counts (n = 6,975) were collected from 862 paddocks spread over 102 farms in the North Island of New Zealand. Historical and projected climate data were obtained from the National Institute of Water and Atmospheric Research. The spore count dataset was merged with climate data from corresponding locations, incorporating time lags of 1–53 weeks. Linear regression models were fitted for describing crude associations, while random forest models were fitted for determining variable importance and predicting future spore counts.

**Results:**

Mixed-effect linear regression models explained up to 11% of the variance of log-transformed spore counts by a single lagged climate covariate. The best-fit random forest model had a testing accuracy of 80% in classifying low or high FE risk (> 20,000 spores) with an R^2^ value of 43%. The random forest models suggested time-dependent importance of soil temperature at 10 cm depth, solar radiation, potential evapotranspiration, vapour pressure, soil moisture and minimum temperature, while no or weak evidence of variable importance was found for maximum temperature, rainfall, mean sea level atmospheric pressure, relative humidity and wind speed. Over the next 80 years, our model predicted an increase in the seasonal mean spore counts in the study farms by a mean of 17% (min 6, max 30%) under the high-end greenhouse gas emission scenario (representative concentration pathways (RCP) 8.5). Every decade was associated with an increase in the probability of high-risk spore counts (> 20,000) by 14–22% for the moderate to high emission scenarios (RCP 4.5–8.5). The model indicated increased peak spore counts across most regions over the next 80 years. Specifically, the entire North Island and three districts in the South Island were projected to have high mean peak spore counts by 2100.

**Conclusions and clinical relevance:**

These findings could be used to target high-risk areas to implement mitigation or adaptation measures for FE. In addition, the study highlights the value of ecological data for forecasting environmental disease risks to enhance preparedness for climate change.

## Abbreviations

FE: Facial eczema
ID: Identifier
ML: Machine learning
MSLP: Mean sea level atmospheric pressure
NIWA: National Institute of Water and Atmospheric Research
PET: Penman potential evapotranspiration
RH: Relative humidity
RCP: Representative concentration pathway
SM: Soil moisture
SR: Solar radiation
T_earth_: 10 cm earth (soil) temperature
T_max_: Maximum ambient temperature
T_min_: Minimum ambient temperature
VP: Vapour pressure
VCSN: Virtual Climate Station Network

## Introduction

Climate change poses a significant threat to global health. Emerging evidence indicates that most major disease pathogens are sensitive to climate change through various pathways, which can influence the geographical distribution and severity of diseases (McIntyre *et al*. 2017; Mora *et al*. 2022). However, modelling the impact of climate on disease is challenging due to the complexity of disease ecology and the extensive data requirements needed to capture these patterns. Machine learning (ML) has emerged as a potential tool to address these challenges by identifying climate-disease relationships and forecasting disease risks under climate change scenarios. ML has advantages in handling numerous covariables with non-linear associations, multicollinearity issues and spatial autocorrelation, all of which are typical of ecological data, without making prior assumptions about the structure of data. In veterinary epidemiology, ML techniques have demonstrated considerable potential for analysing large, high-dimensional epidemiological or ecological datasets (Cutler *et al*. 2007; van der Heide *et al*. 2019; Andraud *et al*. 2021). ML is particularly suitable for climate-sensitive disease modelling, given its ability to integrate diverse climate variables to predict disease patterns (Yeh *et al*. 2023; Ssebyala *et al*. 2024). By training models on historical data, ML algorithms can learn the relationships between environmental variables and disease occurrence, providing insights and forecasts for future risk scenarios in the multi-disciplinary fields, without the need for extensive human input and assumptions (Murdoch *et al*. 2019).

This study aimed to develop data-driven ML models to predict the impacts of climate change on disease, using facial eczema (FE) as a case study. FE, or pithomycotoxicosis, is a seasonal disease of grazing livestock that is considered highly sensitive to climate conditions (Brook 1964; Di Menna *et al*. 2009; Phillips *et al*. 2023). FE has historically been a well-recognised problem for grazing sheep and cattle farms in the North Island of New Zealand in summer and autumn seasons (Dawson and Laven 2007; Di Menna *et al*. 2009; Mitchell *et al*. 2011), with an estimated annual cost of around NZD $332 million (Acland and McIvor 2023). Although FE was first identified and is most frequently reported in New Zealand, occurrence of FE has been reported worldwide (Di Menna *et al*. 2009), including recent reports from Europe (Fernández *et al*. 2021; De las Heras *et al*. 2022; Dijkstra *et al*. 2022). FE is caused by mycotoxin sporidesmin, produced by spores of the fungus *Pithomyces chartarum* (Percival and Thornton 1958), later classified as *Pseudopithomyces chartarum* (Ariyawansa *et al*. 2015). The fungus grows on the dead and dying matter at the base of the ryegrass pasture sward (Brook 1963). Abundant grass debris combined with warmer temperatures and rainfall, or humid conditions, were considered as contributing factors for higher spore counts (Brook 1964). When grazing animals ingest grass contaminated with sporidesmin, the toxin accumulates in the bile ducts and causes liver dysfunction, leading to various symptoms including photosensitivity, skin lesions, liveweight loss, reproductive failure, decreased milk production, and death (Cuttance *et al*. 2021).

There is limited understanding of FE incidence and mortality, due to challenges in diagnosis, its chronic nature, and the presence of subclinical cases (Cuttance *et al*. 2021). However, it is generally understood that spore counts correlate with the toxin responsible for FE and the severity of FE liver damage (Smith *et al*. 1965). Although exposure to spores does not necessarily lead to disease, as other factors, such as toxin production, animal intake and resistance, can modify this relationship, spore counting is a primary method for monitoring FE risk on pastures, serving as a decisionmaking tool for farmers implementing control measures, such as zinc supplementation (Smith and Embling 1999; Cuttance *et al*. 2016, 2017).

According to New Zealand climate projections, an extension of warm and moist conditions is likely to occur across many areas of New Zealand (Ministry for the Environment 2018). Because these conditions would be optimal for fungal growth and sporulation, there is a growing concern that the risk of FE may increase in future in New Zealand (Di Menna *et al*. 2009; Dennis *et al*. 2014). However, previous research on FE risk prediction relied on a limited range of climate variables (Crawley and Woolford 1965; Parle 1967; Dennis *et al*. 2014) or pre-assumed relationships with climate predictors (Phillips *et al*. 2023). These studies were hindered by the lack of systematically collected, longitudinal field data. In addition, while the associations with spore counts and immediate temperature, rainfall and humidity are well-documented, fewer studies have explored the associations with a range of other climate variables for longer time periods. Using longitudinal spore count data collected for secondary purposes between 2010 and 2017, the objectives of this study were firstly, to determine the importance of 11 climate variables in predicting *P. chartarum* spore counts in the subsequent 1–53 weeks, and secondly, to forecast the potential future risk of FE over the next 80 years using spore counts as risk indicators and the projected climate data for New Zealand.

## Materials and methods

### Data

This study used the spore count data collected by Veterinary Enterprises Group (VetEnt, Te Awamutu, NZ) and climate observation data and climate projection data provided by the National Institute of Water and Atmospheric Research (NIWA, Wellington, NZ). The data was processed using R 4.2.2 (R Foundation for Statistical Computing, Vienna, Austria) unless otherwise specified. In this study, we use the terms “prediction” to denote the process of estimating spore counts using the climate data within our model framework, and “projection” specifically for future climate data, indicating the representation of future climate scenarios.

#### Spore count data

Spore count data were obtained from seven veterinary clinics belonging to the same veterinary group located in seven territorial authorities in the North Island of New Zealand: Waipā, Ōtorohanga, Waitomo, Ruapehu, Gisborne, Wairoa, and Hastings. These clinics routinely produce reports of spore counts as a service to their clients for FE risk management. The sampling date ranged from 15 December 2010–3 March 2017, which covered seven FE seasons (2010/11–2016/17), where an FE season is defined as starting 1 December and ending 30 June in the following calendar year. The time period corresponds to the New Zealand summer and autumn. The sampling seasons varied by clinics, as detailed in a Table 2 footnote.

Pasture samples were collected from multiple paddocks from each monitoring farm, typically at a weekly interval, although the interval varied by farm and season. For each paddock, 20 g of pasture from 10 sites within a paddock (200 g per paddock) was retrieved. Spore counts were determined by the wash method, where 60 g of grass sample was washed in 600 mL of water, and spores in a sample of the wash water were counted using a microscope at 100x magnification over the grids of a haemocytometer slide.

**Figure 1:**
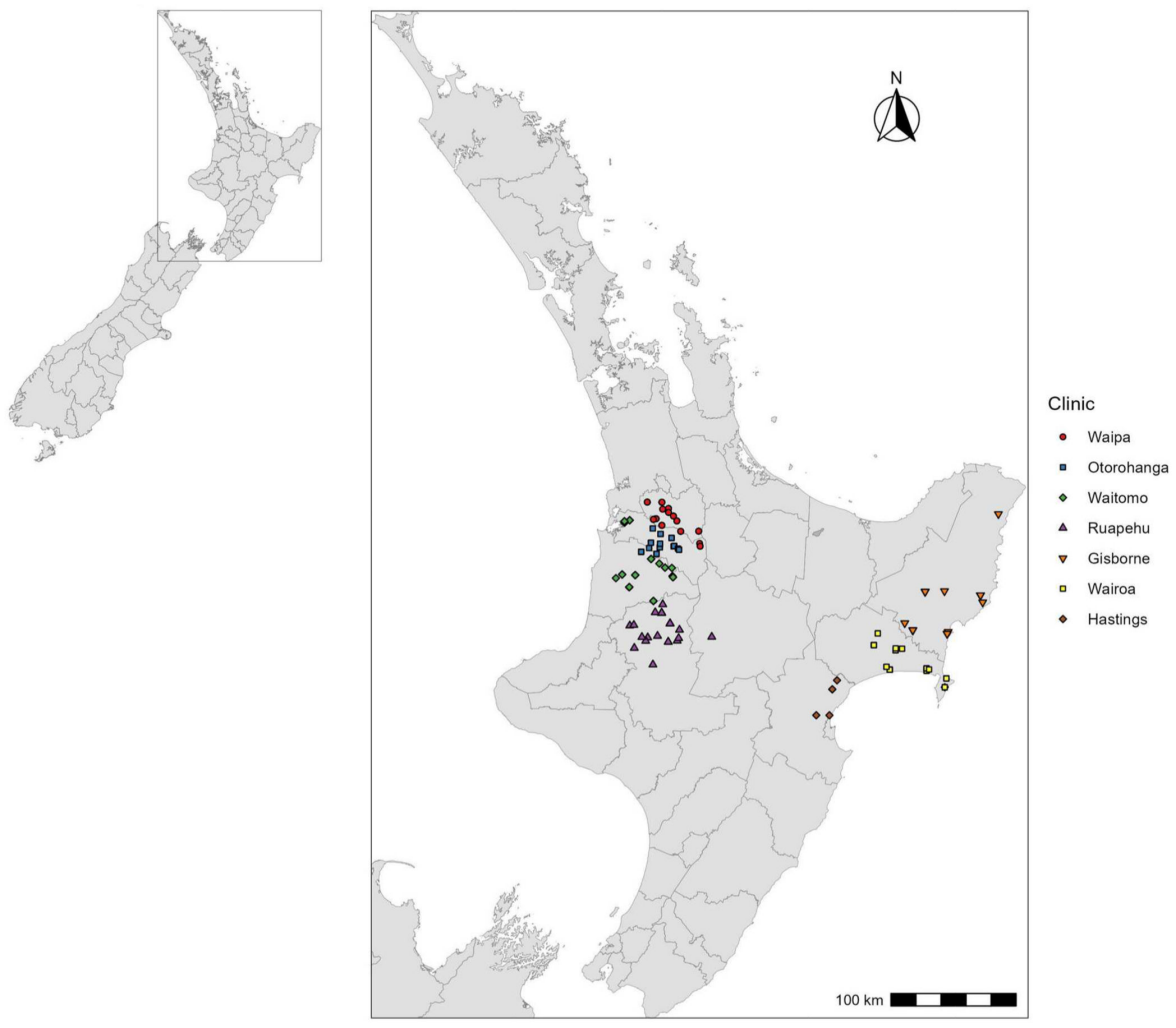
Point locations of 102 farms in the North Island of New Zealand where pasture *Pseudopithomyces chartarum* spore counts were measured between 15 December 2010 and 3 March 2017 and subsequently used in a study to model the effects of climate change on spore count. The colours and shapes of the points represent the veterinary clinics which collected spore samples. For interpretation of the coloured elements in this figure, the reader is referred to the online version of this article.

Spore count data for individual farms were extracted from the report forms into an Excel (Microsoft; Redmond, WA, USA) spreadsheet by the authors. The dataset comprised a unique farm identifier (ID), paddock ID, date of sampling, spore count and veterinary clinic ID. The farm addresses were provided separately as a physical address or road names. There were 131 sampled farms in total, of which only 102 had sufficient information to determine their exact point location (49 farms), or approximate location, i.e. middle point of the road (41 farms), or centroids of the village/township (12 farms) (Figure 1). The geographical coordinates (latitude, longitude) of the sampled farms were determined by the authors using Google Maps. The sample farms likely included dairy farms and mixed beef and sheep farms. However, there was no data regarding farm types.

#### Climate observation data

The Virtual Climate Station Network (VCSN) historical climate observation data were derived from NIWA (Tait *et al*. 2006). The VCSN data contained interpolated observations of 11 climate variables for VCSN grid points spaced at 0.05° latitude and longitude (approximately 5 km x 5 km) across New Zealand. The nearest VCSN grid points from the point location of the study farms were determined for each farm. In total, 75 unique VCSN grids were identified for the 102 study farms, and the daily climate data between 1 January 2010 and 30 June 2017 were extracted. Among the 75 VCSN grids, 59 contained a single study farm, whereas 16 contained multiple (2–5) farms.

The 11 climatic variables were minimum ambient temperature (T_min_), maximum ambient temperature (T_max_), soil temperature at 10 cm depth (T_earth_), solar radiation (SR), rainfall (Rain), vapour pressure (VP), mean sea level atmospheric pressure (MSLP), relative humidity (RH), Penman potential evapotranspiration (PET), soil moisture (SM) and wind speed (Wind) (Table 1).

While previous studies have primarily focused on short-term associations between weather and sporulation, we hypothesised that there are long-term links between climate and spore counts, due to the time required for growth of pasture and dissemination, germination, growth, and sporulation of the fungus (Mitchell *et al*. 2011). The duration of this lag is likely to vary; it could be several days or weeks for fungus sporulation, and several months or years for pasture growth and maturation. In this study, we designed the models to examine both the short- and long-term effects of 11 climate variables on spore counts, considering time lags ranging from 1–53 weeks prior to the date of spore counting. A week was selected as an optimal interval, balancing the noise and complexity of daily data and the oversimplification of monthly data. For instance, rainfall with a 1-week lag was computed as a mean daily rainfall between Day −1 and Day −7, and a 2-week lag between Day −8 and Day −14, counting back from the day of spore counting. To explore the potential associations with spore counts, we included all 11 available climate variables in our model. A total of 583 lagged climate covariates (11 climatic variables x 53 weeks) were computed for each sampling date and farm and merged with the spore count data by the date of records and location.

#### Climate projection data

Projected daily climate data across New Zealand from 1 January 2006–31 December 2100 from six climate models were obtained from NIWA in the Network Common Data Form file format. The details of the models for climate change projection can be found in their report (Ministry for the Environment 2018). Briefly, six global climate models from the Coupled Model Inter-comparison Project, Phase 5, were used to provide baseline conditions for six corresponding regional climate models, which simulated data for 5 × 5 km VCSN grids for New Zealand (Sood 2014). The six models were the Beijing Climate Center Climate System Model version 1.1 (Wu *et al*. 2019), the Community Earth System Model version 1.0 with Community Atmospheric Model version 5 (Meehl *et al*. 2013), the Geophysical Fluid Dynamics Laboratory Climate Model version 3 (Griffies *et al*. 2011), ModelE2 version of the Goddard Institute for Space Studies General Circulation Model (Schmidt *et al*. 2014), the Hadley Centre Global Environmental Model version2 (Collins *et al*. 2011) and the Norwegian Earth System Model version 1 (Bentsen *et al*. 2013). All six models were used for spore count prediction to consider a range of uncertainties and variability in climate projections.

The processing of Network Common Data Form files was conducted using Climate Data Operators version 1.9.8 (Schulweida 2023) and R on New Zealand eScience Infrastructure (NeSI), a high-performance computing environment. There were only seven variables available in the projection data: T_max_, T_min_, SR, Rain, MSLP, RH and Wind (Table 1). For T_max_, T_min_, and Rain, the “linked empirical modelled and observed distribution” correction method was applied to reduce systematic biases and correct distortions in the distribution of the modelled data, to improve agreement with observations (Sood 2014). The correction was not available for SR, MSLP, RH and Wind, and hence the raw predicted data were used (Table 1).

For each of the six regional climate models, two datasets were prepared. The first dataset was for the 75 VCSN grid points matching the study farm locations. The climate projection data between 1 January 2006 and 31 December 2100 were processed into weekly means and merged with farm ID and restructured to the format suitable for model prediction, including 371 lagged climate covariates (7 climatic variables for which projection data were available x 53 weeks), farm ID, paddock ID and week number. The second dataset was for the 66 territorial authorities of New Zealand (excluding Chatham Islands). The raster mean values on a weekly basis (weekly mean values among all grids within each boundary) were computed between 1 January 2006 and 31 December 2100. The data were formatted to include 371 lagged climate covariates and week number, but excluded the identifiers for the farm, paddock, and clinic.

The climate projection was for four emission scenarios described in the *Intergovernmental Panel on Climate Change Fifth Assessment Report*, namely representative concentration pathway (RCP) 2.6, 4.5, 6.0 and 8.5 (van Vuuren *et al*. 2011). They represent a stringent mitigation scenario (RCP2.6), two intermediate scenarios (RCP4.5 and RCP6.0) and a high-end scenario with very high greenhouse gas emissions (RCP8.5).

In summary, projection data for the period 2006–2100 were prepared for six Coupled Model Inter-comparison Project, Phase 5 climate models, four greenhouse gas emission scenarios, and two geographical aggregation levels.

### Statistical analysis

Linear regression models were fitted to determine the crude strength and direction of association between spore counts and each lagged climate covariate. Random forest algorithms were trained to predict future spore counts. The statistical analyses were conducted using R 4.2.2. We used the R packages lme4 version 1.1.35.1 (Bates *et al*. 2015) for mixed effect linear regression models, ranger version 0.16.0 (Wright and Ziegler 2017) for random forest models, and plyr version 1.8.9 (Wickham 2011) and tidyverse version 2.0.0 (Wickham *et al*. 2019) for data processing and visualisation.

#### Linear regression

For the linear regression analysis, the log(x/1000 + 1) transformed spore count (x; spore count per g pasture) was used as an outcome variable. Initially, the normality of the residual distribution was graphically assessed using a simple model with farm as the only fixed effect. After assessing that there were no violations in the assumptions, linear regression models were fitted, using each of the 583 weekly mean lagged climate covariates as an explanatory variable. For each lagged climate covariate, a univariate model with a single lagged climate covariate as a fixed effect and farm and paddock as random effects (583 models in total) was fit:

$$Y_{ijk}=\beta_{0}+\beta_{X}X_{ijk}+\gamma_{j}+\delta_{jk}+\varepsilon_{ijk}$$

where *Y*_*ijk*_ is the log(x/1000 + 1) transformed spore count for observation *i* in farm *j* and paddock *k, β*_0_ is the global intercept, *X*_*ijk*_ is the lagged weekly mean climate covariate for observation *i* in farm *j*, paddock *k, β*_*X*_ is the slope coefficient, γ_j_ is the farm level random effect for farm *j, δ*_*jk*_ is the paddock level random effect for farm *j*, paddock *k*, and *ε*_*ijk*_ is the residual error for observation *i* in farm *j* and paddock *k*.

The variance explained by farm or paddock was assessed by the intraclass correlation, calculated as the ratio of its variance to the total variances. The crude associations between lagged climate covariates and spore counts were assessed by the estimated coefficients and the marginal R^2^ values.

#### Random forest

Random forest is a ML technique where a collection of classification or regression decision trees are fitted to randomly and independently selected subsets of a dataset (Breiman 2001; Genuer and Poggi 2020). Within a forest, multiple trees are fitted, and a simple majority vote or an average of the predictions among all trees in the forest is taken for the final output.

First, we examined the performance of three types of random forest models to identify the most suitable model design for the data distribution. The three models differed in how they handled the spore count variable and in the prediction method used:

- a classification random forest model to categorise spore counts as low or high risk of FE using a threshold of 20,000,
- a regression random forest model applied to log(x/1000 + 1)-transformed spore counts, and
- a hybrid model combining a classification random forest to categorise spore counts as 0 or >0, followed by a regression random forest for log(x/1000 + 1) transformed spore counts for positive spore counts.

The explanatory variables for each of the three models were 583 weekly mean lagged climate covariates, clinic ID, farm ID, paddock ID, season ID and number of weeks from the start of the FE season (1 December). Latitude and longitude of farm locations were excluded because they did not improve model performance.

Before training each model, two hyperparameters were optimised by simultaneously varying the values by grid search (node size = 5, 40, 60, 80; sample fraction = 0.550, 0.632, 0.700, 0.800), and the set of parameters that minimised the error rate was used for training and prediction (Probst *et al*. 2019). The number of trees was set to 1,000. For other hyperparameters, the package-calculated default values were used.

We trained the three models using a 10-fold cross-validation approach, in which the data were randomly divided into 10 subsets. In each iteration, each of the three models was trained using nine of these subsets combined into a single training dataset, leaving the remaining one subset as a testing dataset. The process was repeated for 10 iterations.

To evaluate the model performance, performance metrics were calculated for training and testing datasets, respectively, for each iteration, and then averaged across 10 iterations. A 2 × 2 confusion matrix was created for model-predicted values using a spore count of 20,000 as a cut-off, counting true positive (TP), false positive (FP), true negative (TN) and false negative (FN) for both training and testing data. The performance of the models was measured by accuracy (accuracy = (TP + TN)/(TP + FP + TN + FN)), as well as sensitivity (Se = TP/(TP + FN)) and specificity (Sp = TN/(TN + FP)). In addition, the performance of the regression model and the hybrid model was evaluated by the variance explained (R^2^) and mean squared error. The best model type was determined based on the highest mean accuracy by cross-validation on testing data. Variable importance was measured by the variance of importance scores using the best fit random forest model. The variable importance was standardised to range from 0 to 1, with 1 being the highest importance.

To assess the model’s performance at extended locations across New Zealand, the selected best model type was retrained using the same spore count data collected between 2010 and 2017, but using 371 lagged climate covariates for which the projected climate data were available (T_max_, T_min_, SR, Rain, MSLP, RH and Wind) plus week number, excluding clinic ID, farm ID, paddock ID and season ID. The model was trained through 10-fold cross-validation, using (i) nine randomly selected subsets of all data (reference), (ii) nine subsets of data from Waikato (Waipā, Ōtorohanga, Waitomo and Ruapehu) and (iii) nine subsets of data from East Coast (Gisborne, Wairoa, and Hastings). For (ii), the model was tested using a subset of East Coast data. Conversely, for (iii), the model was tested using a subset of Waikato data. The process was repeated for 10 iterations, and the mean and 95% CI of the model performance were assessed.

### Prediction of spore counts

The projected climate data between 2006 and 2100 for RCP2.6, 4.5, 6.0 and 8.5 for the six climate models was applied to the random forest prediction model. The prediction was made for 102 study farms, using the most frequently sampled paddock, as well as for zonal means, i.e. the mean values of all raster cells within each administrative region (66 territorial authorities). Instead of raw grids (n = 11,491), zonal means at the territorial authority level (n = 66) were used to reduce computational burden, as such high-resolution detail was not required for country-wide prediction. Moreover, the geographical coverage of the training data was considered insufficient to reliably capture variation at the grid scale.

For each combination of RCP projection (n = 4), climate model (n = 6) and study farm (n = 102), we evaluated the ratio of the seasonal mean spore counts relative to the 2006–2020 average as a baseline. In addition, we evaluated the temporal change in five summary outcomes; the predicted seasonal mean spore counts (December to June), the seasonal peak spore counts, the probability of spore counts exceeding 20,000 for at least 1 week during the season, the first week of the season when predicted spore count exceeded 20,000, and the mean number of weeks when predicted spore count exceeded 20,000. For each outcome, a temporal trend of the outcome variable was examined by a mixed-effect linear regression model or a mixed-effect logistic regression model using year, RCP and an interaction term of the two as covariates and farm as a random effect. We excluded climate model as a predictor, so that the results reflect an ensemble average, incorporating variation across climate models. For each RCP, the coefficients or OR of the year term were assessed as an indication of the size of the effect of climate change.

For indicative FE risk forecasting for the whole of New Zealand, for each RCP, the predicted weekly spore counts for territorial authorities were averaged across climate models, and the period mean was computed for five periods: 2006–2020, 2021–2040, 2041–2060, 2061–2080 and 2081–2100. Choropleth maps were generated to visualise the spatio-temporal changes in the predicted potential FE risks over the 80 years.

## Results

There were 6,975 spore count observations from 862 paddocks from 102 farms between 2010 and 2017, with sampling coverage varying by clinic. The spore counts (spores/g) ranged from 0 to 10,000,000 with an overall median of 10,000 and a mean of 36,937 (Table 2). The distribution of the spore counts was highly right-skewed and zero-inflated, with 37% (2,580/6,975) of the data values being 0, and 5% of the data ranging from 150,000–10,000,000. There was a high variability in spore counts between time points, between paddocks within farms, between farms, and between seasons. In general, the average spore count increased from January, peaked around March and then decreased. The season 2015/16 had particularly higher spore counts than the other FE seasons, with a median of 40,000.

With linear regression models, the variance in log(x/1000 + 1)-transformed spore counts explained by a single lagged climate covariate was 0.0–11.0% (mean 2.6%) for models with paddock and farm as a random effect. Using paddock nested within farm as random effects, a mean of 20.8% (min 18.1, max 23.1)% of the variance was attributed to paddock and 9.9 (min 8.6, max 13.4)% to farm, indicating the paddock level contributed more to the total variability than the farm level.

**Figure 2:**
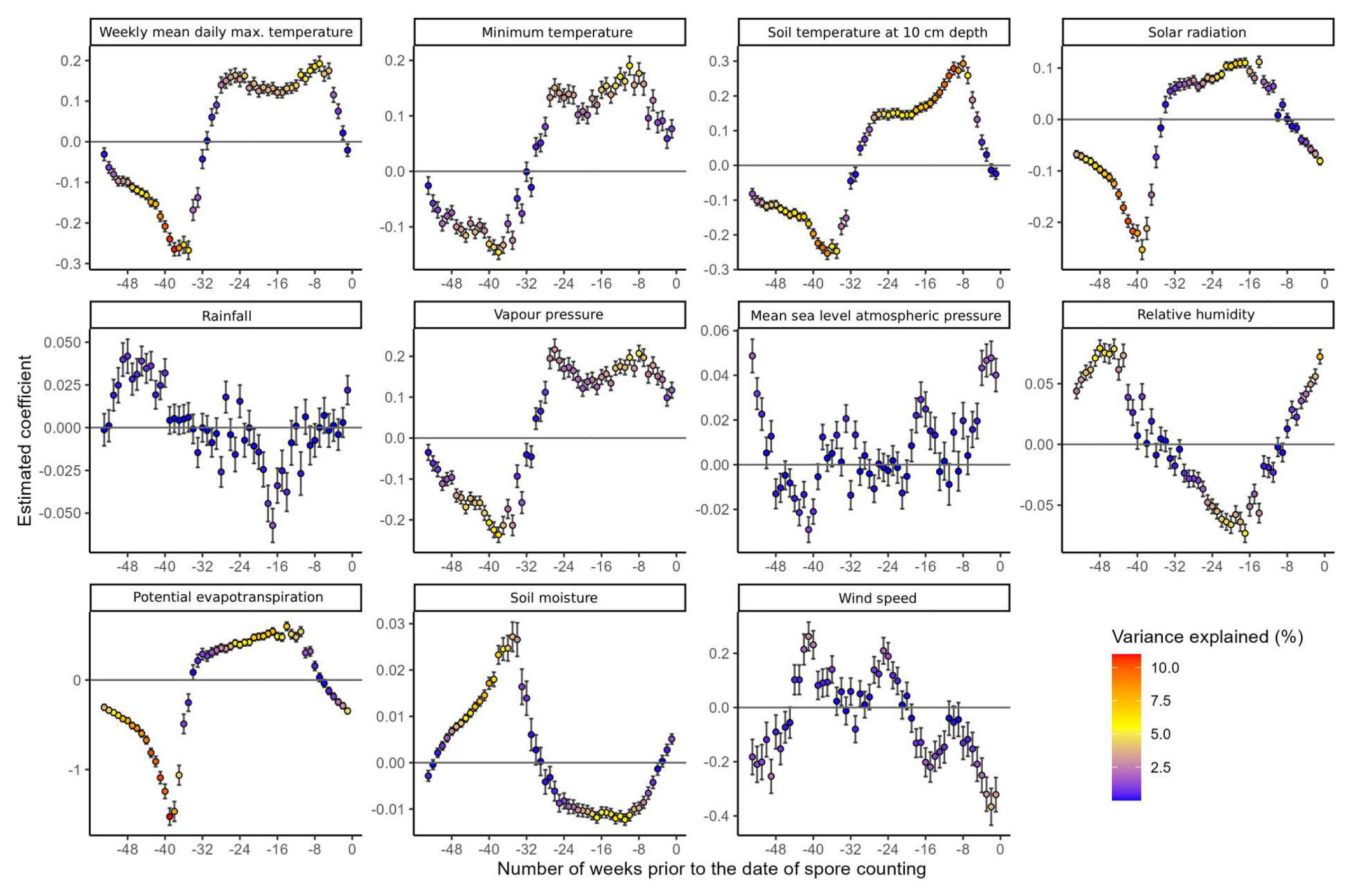
Crude association between climatic covariates lagged from 0–53 weeks prior to count estimation and *Pseudopithomyces chartarum* spore count (on the scale of log(x/1000 + 1)) for farms from the North Island of New Zealand estimated by mixed-effect linear regression models (based on a single climate covariate) with farm and paddock as random effects. Points represent estimated regression coefficients and error bars 95% CI. The facets show the 11 climatic variables, and x-axis the time lag (weeks) from sampling date. The gradient colours of points indicate marginal R^2^ values. For interpretation of the coloured elements in this figure, the reader is referred to the online version of this article.

Based on the variance explained by the random effect models where paddock was nested within farm, SR, PET, T_max_ and T_earth_ 35–50 weeks prior to spore counting indicated the strongest explanatory power for spore counts (Figure 2). The direction of the association for these explanatory variables was consistently negative, indicating that lower SR, PET, T_max_ and T_earth_ was associated with higher spore counts 35–50 weeks later. Following these explanatory variables, T_earth_ (8–18 weeks prior) and SR (18–20 weeks prior) showed the strongest explanatory power (R^2^) and the direction of the associations were positive. Furthermore, RH in the week before spore counting was positively associated with spore counts.

All three random forest models showed a reasonable fit in classifying low or high spores, with testing accuracies ranging from 76.8% to 80.1% (Table 3). The hybrid model had the highest testing sensitivity (69.0%) in detecting high spore counts, however, the lower testing accuracy (76.8%) and the R^2^ value (21.9%) indicate a potential issue of overfitting. The regression random forest model had the highest testing accuracy (80.1%) with an R^2^ value of 43.3%, demonstrating a better ability to explain the data variability.

**Figure 3:**
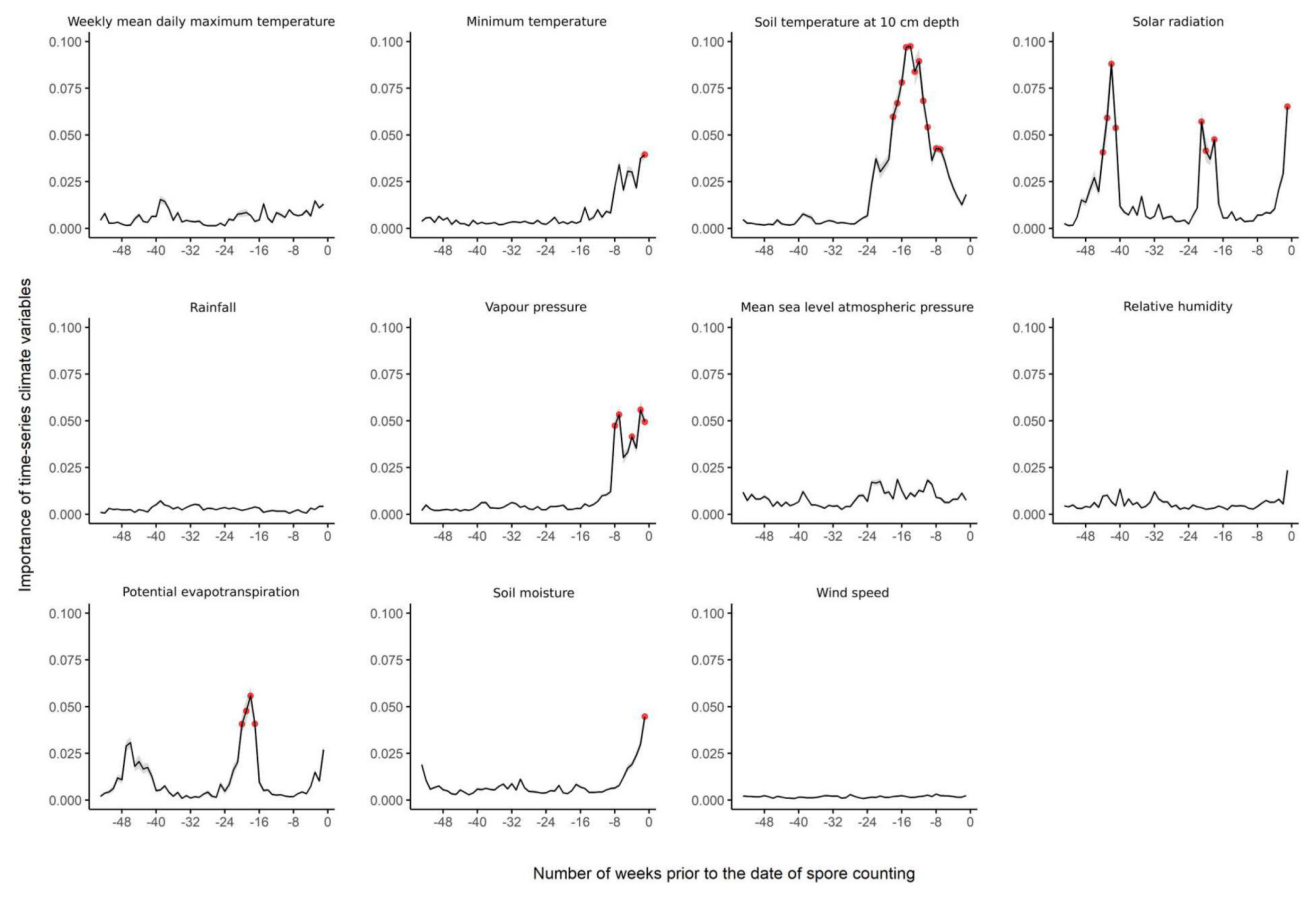
Mean standardised variable importance for 11 climatic variables by lagged week, from a regression random forest model used to predict *Pseudopithomyces chartarum* spore counts for farms from the North Island of New Zealand. Top 5% most important variables are highlighted with red points, and the grey shaded area is the 95% CI. For interpretation of the coloured elements in this figure, the reader is referred to the online version of this article.

With the regression random forest model, paddock ID and farm ID were the most important variables with the standardised variable importance of 1.00 and 0.21, respectively. The standardised variable importance of lagged climate covariates by week is shown in Figure 3. T_min_, T_earth_, SR, VP, PET, and SM showed clear temporal patterns characterised by distinct peaks; within a week before spore counting (T_min_, SR, VP, SM), 7 weeks (VP), 14 weeks (T_earth_), 18 weeks (PET), 21 weeks (SR) and 42 weeks (SR) before spore counting. The variable importance of T_max_, Rain, MSLP, RH and Wind was relatively low or had no clear temporal patterns.

We selected the regression random forest as our prediction model. When the prediction model was trained with a limited set of lagged climate covariates (n = 371) without clinic ID, farm ID, paddock ID and season ID, the mean accuracy was 78.6 (95% CI = 77.8–79.4)% with an R^2^ value of 39.7 (95% CI = 37.4–41.9)% on testing data, which was comparable to the full model (Accuracy = 80.1%; R^2^ = 43.3%). However, when the same model was trained with Waikato data and tested on East Coast data, the mean accuracy decreased to 65.1 (95% CI = 63.5–66.7)%, with an R^2^ value of 12.4%. Similarly, when the model was trained with East Coast data and tested on Waikato data, the mean accuracy was 70.9 (95% CI = 70.0–71.8)%, with an R^2^ value of 10.3%. Acknowledging the potential limitation in accuracy when the model was applied to unknown locations, we proceeded with the prediction for the whole country at the territorial authority level.

**Figure 4:**
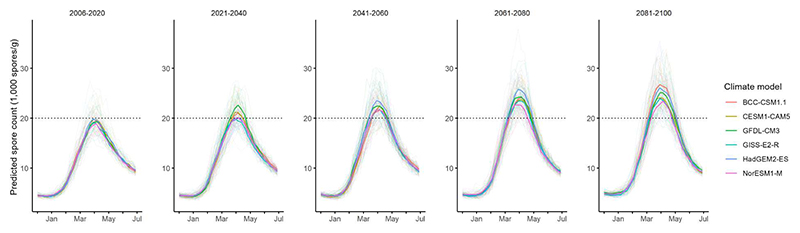
Annual predictions by month from a random forest regression model for the seasonal *Pseudopithomyces chartarum* spore count stratified by time period for an example farm in Waikato, New Zealand for climate projections from six different climate models under Representative Concentration Pathways 8.5, representing a high greenhouse gas emission scenario. The six climate projection models represented by different line colours were the Beijing Climate Center Climate System Model version 1.1 (BCC-CSM1.1); the Community Earth System Model version 1.0 with Community Atmospheric Model version 5 (CESM1-CAM5); the Geophysical Fluid Dynamics Laboratory Climate Model version 3 (GFDL-CM3); ModelE2 version of the Goddard Institute for Space Studies General Circulation Model (GISS-E2-R); the Hadley Centre Global Environmental Model version2 (HadGEM2-ES) and the Norwegian Earth System Model version 1 (NorESM1-M). The period mean across years is shown by bold lines. The lower limit of high risk of facial eczema (20,000 spores/g) is indicated by dotted lines. For interpretation of the coloured elements in this figure, the reader is referred to the online version of this article.

**Figure 5:**
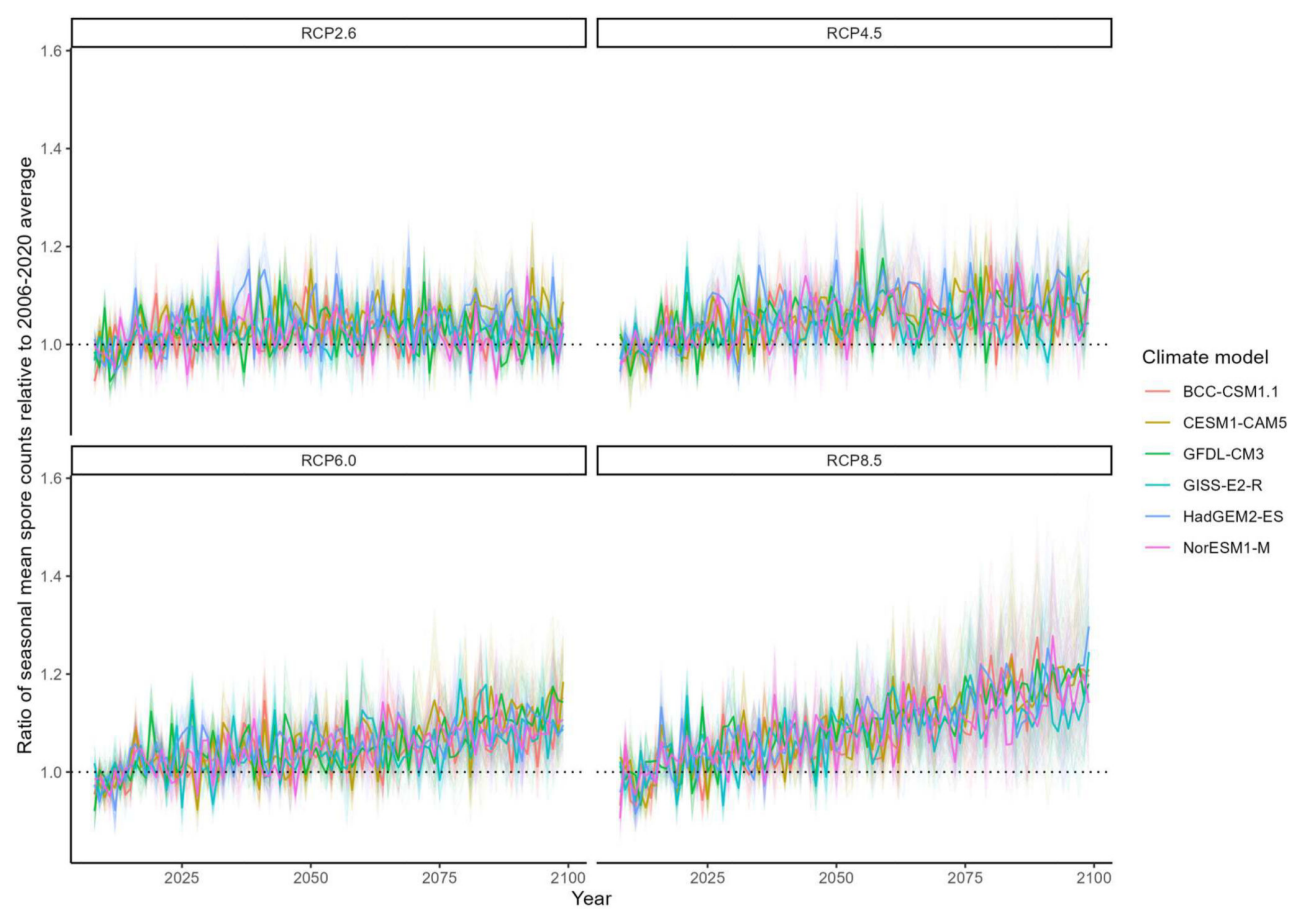
Predicted change, from a random forest regression model, in seasonal mean *Pseudopithomyces chartarum* spore counts between 2021 and 2100 expressed as ratios to the 2006–2020 average, for 102 study farms in the North Island of New Zealand for climate projections from six different climate projection models under four representative concentration pathways (RCP), ranging from a stringent mitigation scenario (RCP2.6) to a high-end scenario with very high greenhouse gas emissions (RCP8.5) For identification of the six climate projection models represented by different line colours, please see the caption for Figure 4. Farm-level season mean spore counts are shown by thin lines, and the season mean across farms is shown by bold lines. The dashed lines (ratio = 1.0) indicate no change from the baseline. For interpretation of the coloured elements in this figure, the reader is referred to the web version of this article.

Using the projected local climate conditions for the four emission scenarios, weekly spore counts were predicted for the study farms from 2006 to 2100. In general, the seasonal mean and peak spore count increased over time in the higher emission scenarios, but the extent of the change varied by farms. Figure 4 shows the predicted seasonal spore counts for a particular farm in Waikato from 2006 to 2100 for the highest climate emission scenarios (RCP 8.5). There was an increasing trend in the predicted peak spore counts as well as the duration of high-risk FE over the 80 years. Predicted spore counts for this farm for other RCP scenarios are provided in Supplementary Figure 1. The predicted increase in the seasonal mean spore counts from 2021 to 2100 compared with the 2006–2020 average, at the farm level and averaged across farms, is shown in Figure 5. For RCP 2.6, there was no apparent long-term trend, with the ratio fluctuating around 1, indicating no change in the seasonal spore counts from the baseline. For RCP 8.5, the farm-level ratios generally increased linearly over the 80 years, with an increasing variability (min 0.925, max 1.570). The mean ratio across farms in 2081–2100 for RCP 8.5 was 1.167 (min 1.056, max 1.297), indicating a 16.7% increase in the mean spore counts compared with the 2006–2020 average. Table 4 compares the estimated effects of time on the spore count prediction outcomes for study farms. The effects of time on the outcomes were all significant regardless of their magnitude, due to large data size. The magnitude of the effects of time was generally higher for higher emission scenarios. For RCP 8.5, a decade was associated with an increase in the predicted seasonal peak and mean spore counts on the log scale by 10.8 × 10^−3^ and 16.6 × 10^−3^, respectively, equivalent to approximately 1.08% and 1.66% increase per decade on the original scale (note: the predicted values on original scale (spore count/g pasture) were back transformed as x = 1000[exp(y) −1], where y is the predicted values on the log scale). Every 10 years was associated with an increase in the predicted probability of exceeding 20,000 spore counts by 14.2–22.0% for RCP 4.5, 6.0 and 8.5. The effect of time on the duration or the start of >20,000 spore counts was negligible; the largest effect was predicted for RCP 4.5, in which the period of >20,000 spore count started 5.7 × 10^−2^ weeks (0.4 days) earlier, and extended by 4.8 × 10^−2^ weeks (0.3 days) each decade.

Using the zonal mean climate projection, the spore counts were predicted for territorial authorities between 2006 and 2100. The predicted mean peak spore counts for five periods for the four RCP and the percentage difference from the baseline (2006–2020) are shown in Figure 6. During 2006–2020, areas where predicted mean peak spore counts exceeded 15,000 were located in the northern parts of the North Island, including Northland, Auckland, Bay of Plenty and Waikato (Figure 6(A)). No territorial authority had predicted mean peak spore counts of >20,000 during this period. In the next 80 years, the model indicated an increase in the peak spore counts for all territorial authorities for RCP 8.5 by up to 82.8%, except for Northland, where a decrease by up to 15.0% was predicted (Figure 6(B)). In the next 80 years, the areas exceeding predicted mean peak spore counts of 15,000 spread to the whole or most of the North Island for RCP 6.0 and 8.5, and to Nelson in the South Island for RCP 8.5 (Figure 6(A)). In addition, for RCP 6.0 and 8.5, territorial authorities in Bay of Plenty and Waikato exceeded the predicted mean peak spore counts of 20,000 in 2061–2100.

## Discussion

This study developed random forest models using longitudinal *P. chartarum* spore count data to determine the importance of 11 climate variables, and to predict future indicative FE risks for the study locations and for the whole of New Zealand over the next 80 years. Research focused on modelling and forecasting the impact of climate change on diseases is still in its early stages, with a limited available body of knowledge. Nevertheless, the importance of such studies is growing rapidly from the perspective of public health, livestock health, food safety and food security (Yeh *et al*. 2023).

Our final predictive random forest regression model indicated higher spore levels and more frequent and more widespread “danger” spore levels, indicating increasing risks of FE outbreaks in the North Island of New Zealand in the higher emission scenarios (RCP 6.0 or 8.5) over the 80 years. This largely aligned with the previous research (Di Menna *et al*. 2009; Dennis *et al*. 2014; Phillips *et al*. 2023). However, at the finer spatial resolutions, some differences were observed in the predicted high spore count locations between this study and previous research, and among previous studies. These differences in the predictions may be due to differences in the methods, underlying data, data resolution, and the assumptions used, or suboptimal predictive accuracy.

To our knowledge, this was the first study to apply an ML approach to measure the importance of a range of climate variables on *P. chartarum* spore counts for an extended preceding period of up to one year. The random forest models had the advantage of evaluating the importance of all potential lagged climate covariates over this extended time period, and this was not investigated in previous studies. Specifically, the models highlighted the importance of T_earth_, SR, PET, VP and SM in predicting spore counts for up to approximately 10 months prior to FE season. When a regression random forest model was trained using climate variables with shorter lags (1–6 weeks), its predictive performance on the testing data (R^2^ = 42.0%) was slightly lower than the model with longer lags (1–53 weeks; R^2^ = 43.3%). This may in part be explained by climate autocorrelation, where climate variables at longer lags are correlated with those at shorter lags. However, it is likely that climate influences the sporulation process in different timeframes; inclusion of both longer and shorter lag covariates is preferred for comprehensive demographic models under varying climate (Evers *et al*. 2023).

The linear regression models based on single covariates suggested correlation between higher spore counts and lower SR and lower PET during the previous autumn to winter months, and with higher SR and warmer T_earth_ about 2–4 months prior to sampling. This pattern roughly aligned with the periods of high variable importance identified by the random forest model. These delayed effects of climate variables on spore counts might be because the creation of suitable conditions for fungal growth, such as growth of ryegrass or the accumulation of dead pasture litter where fungus would thrive, are influenced by farm type, pasture or stock management practices, slope, aspect, soil type and irrigation. While immediate weather conditions are often documented as critical in the associations with sporulation (Mitchell *et al*. 1961; Smith *et al*. 1965; Di Menna and Bailey 1973), spore counts might be affected by multiple conditions over a period of time, which could include development of the dead litter or a population build up in the preceding winter, spring or summer (Brook 1969; Mitchell *et al*. 2011). For other fungal groups, a few studies have reported associations between fruiting and climate variables with lags of several months or even into the previous year (Kauserud *et al*. 2008; Diez *et al*. 2013). Earlier studies were constrained by data limitations and the lack of suitable analytical techniques, but future studies with more comprehensive data will be needed to further investigate these long-lagged effects of climate variables.

The random forest models also showed the importance of lagged climate covariates in the immediate week prior to sampling, including T_min_, SR, VP and SM. The positive association between spore counts and SM or RH were suggested in past studies (Mitchell *et al*. 1961; Smith *et al*. 1965). Sporulation was also suggested to be conditional on T_min_ (Smith *et al*. 1965; Di Menna and Bailey 1973). Interestingly, rainfall has often been suggested as a risk factor for spore counts (Brook 1964; Smith *et al*. 1965; Phillips *et al*. 2023). However, we found no evidence of association between rainfall and spore counts, using either linear regression models or random forest models. While rainfall provides instant moisture to the soil, SM is influenced by various other factors, including evapotranspiration, plant uptake, soil types, and accumulated weather conditions over time (e.g. previous rainfall, temperature and humidity). Poor correlation between rainfall and humidity or saturation deficit at regional scales was reported, with rainfall not being an adequate surrogate for evaluating the physiological processes of ticks (Alonso-Carné *et al*. 2015). The presence of important meteorological variables other than rainfall to explain spore counts, such as heavy dews, has also been indicated (Di Menna and Bailey 1973). There is limited literature regarding the role of SR in the fungus sporulation. Previously, a field study speculated the role of sunshine on sporulation and spore dispersion (Smith *et al*. 1961), and an experimental study reported that ultraviolet radiation increased sporulation and sporidesmin production (Di Menna *et al*. 1970). Sunlight or ultraviolet light was also suggested to play a role in sporidesmin toxin inactivation (Marbrook and Matthews 1962).

Due to the complexity of the random forest models, reporting and interpreting the relationships can be challenging. In response to growing demands for improved transparency, interpretable ML has become an active area of research, although existing methods remain limited (Murdoch *et al*. 2019). Therefore, we provided the results of the linear regression models as an indication of the direction of the crude associations. The two models are complementary; the random forest model captured complex relationships involving multiple covariates and time lags, whereas the linear regression models provide a crude indication of the direction of association for individual covariates. Although the combined effects and interactions captured by the random forest may not be fully reflected in the regression models, the regression results generally aligned with the key climate covariates identified by the random forest model as well as with previous studies, highlighting the value of using complementary approaches to understand the relationships between climate and spore counts. More studies are needed to understand the roles of various climatic variables in spore counts.

That spore counts were recorded as part of routine monitoring and may have lacked accuracy is a limitation of the study. They are also known to be highly variable between paddocks within a farm, with a poor repeatability of a single spore counting (Cuttance *et al*. 2017), which may have contributed to the unexplained variance in our models. Removing T_earth_, VP, PET and SM had minimal influence on the model performance and predictions, likely due to the presence of other correlated variables working as effective proxies. However, the model had a limited generalisability when applied across different regions. The predictive accuracy of the model was suboptimal (65–71%) when applied to unknown regions. This was not surprising, given that the models were trained on data from the limited geographical coverage. When extrapolated to unknown areas, the models assumed all the non-climatic conditions, including soil types, terrains, flora, and farm management practices, remain the same between the sampled farms and the rest of New Zealand. Given this constraint, we limited its use to interpreting average trends at the territorial authority level, using the zonal mean climate data, rather than projecting high-resolution risk maps for individual grids. Other non-climatic factors such as farm type, slope, aspect, soil type and irrigation were out of scope and not included in this study but could influence spore counts. In future studies, incorporation of additional data from various regions across the country, land use data or altitude, and other environmental variables could be considered to enhance the accuracy of the prediction model.

Using the climate projection data for 2006–2100 for the study farms, our model predicted non-negligible changes in the future spore counts indicative of FE risks for higher emission scenarios. The model prediction indicated that by 2100 the mean spore counts in the study areas are likely to increase by a mean of 15.0% (min 5.6, max 29.7%) compared to the 2006–2020 average for RCP 8.5. Our model also predicted an increase in the probability of high spore counts (> 20,000 spores/g) by 14.2–22.0% each decade for RCP 4.5, 6.0 and 8.5. It should be noted, however, that the prediction did not account for uncertainties in farming practices over the next 80 years, including changes in flora, emergence of competing fungi, treatment options, consumer behaviour, farm management, economy, demography, or landscape, which may be significant.

Data on FE incidence in animals were lacking, limiting our ability to compare the magnitudes of spore counts and the frequency of FE outbreaks in animals. However, there is an accumulation of anecdotal evidence suggesting that in years with higher spore counts, the severity and incidence of FE tend to increase. For example, the high spore counts in 2016 corresponded with the report of high incidence of clinical FE throughout the North Island in the same year (Johnson *et al*. 2017). While *P. chartarum* is widely distributed in sub-tropical countries worldwide (Dingley 1962), not all strains produce sporidesmin. New Zealand has a higher proportion of sporidesmin-producing *Pseudopithomyces* compared with other countries (Collin *et al*. 1998). Given that FE develops in animals by the cumulative intake of sporidesmin, the predicted increase in *P. chartarum* spore counts in the next 80 years indicates a corresponding increase in FE incidence. As a preventive measure, breeding for tolerance to FE for cattle and sheep has been explored (Morris *et al*. 2004), and breeders have been selectively breeding this trait (Amyes and Hawkes 2014). In the long term, selective breeding for FE tolerance in animals in areas predicted with higher spore counts may be a beneficial control method for grazing farms in New Zealand (Di Menna *et al*. 2009).

The current study highlights the importance of systematic recording of longitudinal animal-health-related data. Models trained on incomplete data may introduce bias in analysis. While ML approaches to handle missing values have been developing, considerable limitations remain in existing ML methods to handle missingness in real-world big data (Emmanuel *et al*. 2021). Regarding FE, despite the practice of measuring and reporting spore counts by multiple veterinary clinics and laboratories, historical data were often deleted or overwritten once they served the initial purpose and were not archived for later analyses. For more accurate predictions, it is necessary to train the model with data from various regions across the country. Climate change occurs over many years with high variability from year to year, and decades of longitudinal health-related data are required to analyse the relationships between climate and disease. High-quality clinical data from active surveillance may not be available due to resource constraints. Secondary data, such as syndromic surveillance data from veterinary practices or laboratory submission data, would be valuable, albeit with compromised data quality. To enhance future modelling in the context of climate and health, improving the quality and completeness of health data is paramount. To achieve this, a deeper commitment is required to evolve current data systems to facilitate interdisciplinary collaborations and data sharing across clinics, laboratories, industry, government, epidemiologists and data scientists over extended periods. Fostering a mutual understanding of the data value among stakeholders is essential for effective collaboration. This study demonstrated the value of such data, which may motivate stakeholders to allocate resources for the collection and maintenance of health-related information, to gain insights into climate change preparedness.

## Conclusions

This study demonstrated the use of a ML approach to model the potential impact of climate change on the risk of an environmental disease, using FE as a case study. Our analysis indicated strong time-dependent importance of T_earth_, SR, PET, VP and SM during the 10 months preceding the measured *P. chartarum* spore counts. Our models predicted an increase in the mean spore counts and a higher probability of experiencing high-risk spore counts by 2100 for RCP 6.0 and 8.5. Preventive measures are warranted to mitigate the climate change impacts on FE in high-risk areas in the North Island. This study also demonstrates the importance of collecting and curating longitudinal health data, to be able to build prediction models that are locally accurate.

## Supplementary Material

Supplemental data for this article can be accessed online at https://doi.org/10.1080/00480169.2025.2579134

Suppl 1

## Figures and Tables

**a:**
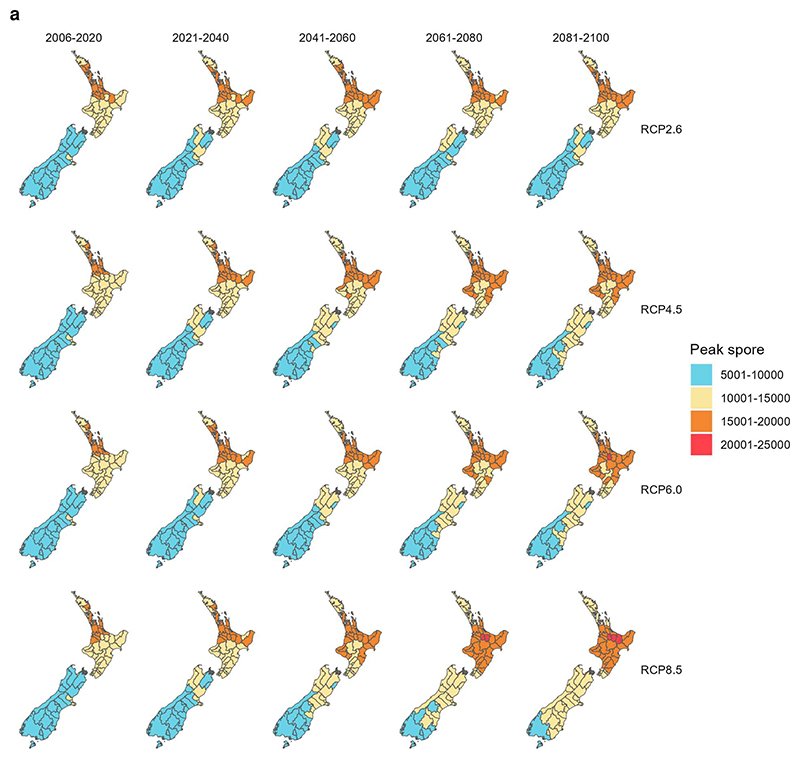


**b:**
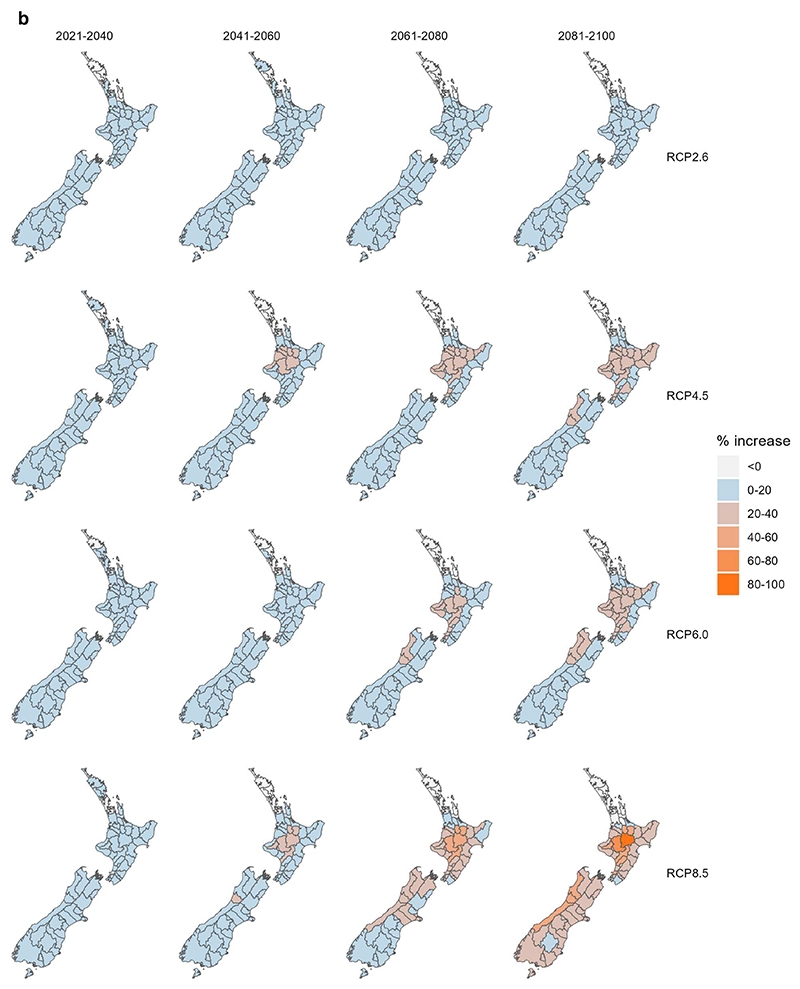


**Table 1:** List of variables in Virtual Climate Station Network (VCSN) historical climate observation data, obtained from National Institute of Water and Atmospheric Research (NIWA), New Zealand, that were available for prediction of spore counts from existing climate data and the availability and format of this data.

Variable name	Unit	Description	Projection data^a^
T_max_	°C	Maximum ambient temperature from 9 am local day.	1
T_min_	°C	Minimum ambient temperature to 9 am local day.	1
T_earth_	°C	Soil temperature at 10 cm depth at 9 am local day.	0
SR	MJ/m^2^	Amount of accumulated global solar radiation from midnight local day.	2
Rain	mm	Total amount of rain from 9 am local day.	1
VP	hPa	Vapour pressure at 9 am local day.	0
MSLP	hPa	Atmospheric pressure reduced to mean sea level at 9 am local day.	2
RH	%	Relative humidity at 9 am local day.	2
PET	mm	24-hour Penman potential evapotranspiration total from 9 am local day.	0
SM	mm	Soil moisture from 9 am local day calculated from rainfall and evapotranspiration^b^	0
Wind	m/s	Mean wind speed at 10 m above ground level over 24 hours from midnight local day.	2

a 0 = unavailable; 1 = bias-corrected data; 2 = uncorrected raw data

b The base value is −150 mm (“permanent wilting point”) based on “soil store capacity”. 0 indicates the soil is at “field capacity” (amount of water held in the soil after the excess has drained away). >0 indicates runoff.

**Table 2:** Summary statistics of *Pseudopithomyces chartarum* spore count observations (x10^3^ spores/g pasture) in 862 paddocks from 102 farms across the North Island of New Zealand, sampled between December 2010 and March 2017 and used in a study to model the effects of climate change on spore count. Data are displayed by clinic, facial eczema season (December to June) and month. Spore counts ≥ 20,000 spores/g indicate a high risk of facial eczema.

	Spore count (x10^3^/g)						Percent of counts			Number of observations
	Minimum	Maximum	95% percentile	Median	Mean		> 0	> 20,000 x10^3^/g		
Overall	0	10,000	150	10	36.9		63.0	31.5		6,975
Clinic^a^										
Gisborne	0	1,920	190	20	55.1		72.1	46.5		810
Ruapehu	0	510	150	10	35.5		64.8	34.3		361
Hastings	0	300	150	20	48.3		79.7	48.4		64
Ōtorohanga	0	920	150	10	33.0		62.6	31.7		1,309
Waipā	0	10,000	170	10	41.9		61.1	28.8		2,010
Waitomo	0	2,160	120	10	30.7		64.0	27.8		1,485
Wairoa	0	610	113	10	25.8		56.4	27.8		936
Season										
2010/11	0	2,160	150	20	46.9		72.3	44.5		512
2011/12	0	80	30	0	7.5		39.4	8.3		277
2012/13	0	340	60	0	12.5		38.8	12.4		1,182
2013/14	0	560	120	10	32.5		67.4	34.5		1,172
2014/15	0	1,010	90	10	22.6		54.2	25.0		1,361
2015/16	0	10,000	310	40	95.1		89.6	61.4		1,268
2016/17	0	490	99	10	22.7		65.9	23.2		1,203
Month										
December	0	0	0	0	0.0		0.0	0.0		12
January	0	190	30	10	6.8		31.6	7.3		1,138
February	0	10,000	130	20	36.4		63.0	28.0		1,918
March	0	2,160	200	20	50.6		74.3	42.6		2,040
April	0	1,010	190	10	47.1		75.0	43.6		1,402
May	0	770	100	0	23.9		56.4	21.6		450
June	0	50	36	0	10.7		46.7	13.3		15

a Waitomo and Wairoa clinics were sampled in all seven seasons. The other clinics started sampling in the 2013/14 season (Waipā, Ōtorohanga and Ruapehu), missed the 2011/12 season (Gisborne), or were only sampled in the 2010/11 season (Hastings).

**Table 3:** Mean (95% CI) performance parameters of three random forest models for predicting *Pseudopithomyces chartarum* spore counts using 583 lagged climate covariates, clinic identifier (ID), farm ID, paddock ID, facial eczema season ID and week number (n = 6,975 spore count observations).

	Binomial model	Regression model	Hybrid model
Model description	Classification forest	Regression forest	Classification forest + regression forest
Outcome variable	< 20 or > 20 (x10^3^ spores/g)	log(x/1000 + 1)-transformed spore count (x; per g pasture)	Classification: 0 or > 0 Regression: log(x/1000 + 1)-transformed spore count (x; per g pasture)
Hyperparameters			
Target node size	80	40	5
Sample fraction	0.555	0.800	0.800
Performance^a^			
Training data			
Accuracy (%)	85.3 (85.2–85.4)	84.7 (84.6–84.8)	88.2 (88.1–88.3)
Sensitivity (%)	64.7 (64.1–65.3)	66.6 (66.3–66.9)	83.1 (82.9–83.4)
Specificity (%)	85.4 (85.2–85.6)	93.1 (93.0–93.2)	90.5 (90.4–90.6)
R^2^ (%)		65.8 (65.6–66.0)	75.5 (75.3–75.6)
MSE^b^ (x10^3^)		3.20 (3.18–3.22)	2.35 (2.34–2.36)
Testing data			
Accuracy (%)	79.9 (78.9–80.9)	80.1 (79.2–81.0)	76.8 (75.6–78.0)
Sensitivity (%)	54.5 (51.5–57.5)	57.1 (54.5–59.8)	69.0 (66.9–71.0)
Specificity (%)	81.4 (80.0–82.7)	90.6 (89.9–91.4)	80.5 (79.2–81.7)
R^2^ (%)		43.3 (41.3–45.3)	21.9 (17.9–25.8)
MSE^b^ (x10^3^)		6.88 (6.50–7.26)	14.41 (12.73–16.09)

a The cut off for sensitivity, specificity and accuracy was 20,000

b Mean squared errors (MSE) are transformed back to the original scale

**Table 4:** Summary of the results from the regression forest model exploring the relationship between predicted changes in *Pseudopithomyces chartarum* spore count outcomes (per g pasture) for study farms from the North Island of New Zealand, over the projected changes in climate between 2006 and 2100 under four different representative concentration pathways (RCP) scenarios, representing low (RCP2.6), moderate (RCP4.5 and RCP6.0) and high (RCP8.5) levels of greenhouse gas emissions. For the outcomes relating to spore counts, start week for the first elevated (> 20,000 spores/g) and mean number of weeks with a spore count > 20,000 spores/g, the coefficients represent the change (with 95% CI) in the outcome over a 10-year time span. When the outcome is the odds that the spore count will >20,000 spores/g for at least 1 week, the value represents the OR (and 95% CI).

Outcome	Scale	RCP2.6			RCP4.5			RCP6.0			RCP8.5	
		Effect	Sig.^c^		Effect	Sig.^c^		Effect	Sig.^c^		Effect	Sig.^c^
Seasonal peak spore counts (transformed)^a^	x10^–3^	3.5 (2.9–4.0)	***		10.0 (9.4–10.5)	***		8.3 (7.7–8.9)	***		10.8 (10.2–11.3)	***
Seasonal mean spore counts (transformed)^a^	x10^–3^	2.3 (2.1–2.5)	***		6.7 (6.5–6.9)	***		9.4 (9.3–9.6)	***		16.6 (16.4–16.8)	***
First week of spore counts > 20,000^b^	x10^–2^	–1.8 (–3.2 to –0.5)	**		–5.7 (–7.0 to –4.4)	***		–2.7 (–4.1 to –1.4)	***		2.0 (0.7–3.3)	**
Mean number of weeks with spore counts > 20,000	x10^–2^	1.8 (1.1–2.5)	***		4.8 (4.1–5.5)	***		4.0 (3.3–4.7)	***		3.4 (2.7–4.1)	***
Odds of spore counts > 20,000	Ratio	1.042 (1.039–1.045)	***		1.220 (1.216–1.224)	***		1.142 (1.12–1.164)	***		1.145 (1.119–1.171)	***

a Spore counts (per g pasture) are on the scale of log(x/1000 + 1)

b Every 10 years was associated with an earlier start of high-risk season (> 20,000 spore counts) by 0.018 weeks (3 hours) for RCP2.6

c Significance (Sig.)

*** <0.001

** <0.01
