# Supplementary material for: Machine learning for predicting climate change impacts on Pseudopithomyces chartarum spore counts: a risk indicator of facial eczema
Source: N Z Vet J. Author manuscript; Available in PMC 2026 Jul 14. (PMC7619234; doi:10.1080/00480169.2025.2579134)
Supplement: Suppl 1 [file EMS212925-supplement-Suppl_1.pdf]

Machine learning for predicting climate change impacts on *Pseudopithomyces chartarum* spore counts: a risk indicator of facial eczema

M Wada[a§] O Sagarasaeranee[a,b], N Cogger[a], J Marshall[c], E Cuttance[d,e], G Macara[f], A Sood[f] and E Vallee[a]

[a] EpiCentre, Tāwharau Ora – School of Veterinary Science, Massey University, Palmerston North, New Zealand
[b] Department of Livestock Development, Ministry of Agriculture, Bangkok, Thailand
[c] School of Mathematical and Computational Sciences, Massey University, Palmerston North, New Zealand
[d] VetEnt, Te Awamutu, New Zealand
[e] EpiVets Ltd, Te Awamutu, New Zealand
[f] National Institute of Water and Atmospheric Research, Wellington, New Zealand
[§]Corresponding author: Email: m.wada@massey.ac.nz

The content of this supplementary information has not been edited. All risk and liability rest with the authors.

Supplementary Figure 1. Predicted seasonal *Pseudopithomyces chartarum* spore count stratified by time period for an example farm in Waikato in the North Island of New Zealand for four Representative Concentration Pathways (RCP). The six climate models are represented by line colours. The period mean across years is shown by bold lines. The lower limit of high risk of facial eczema (20,000 spores/g) is indicated by dotted line.

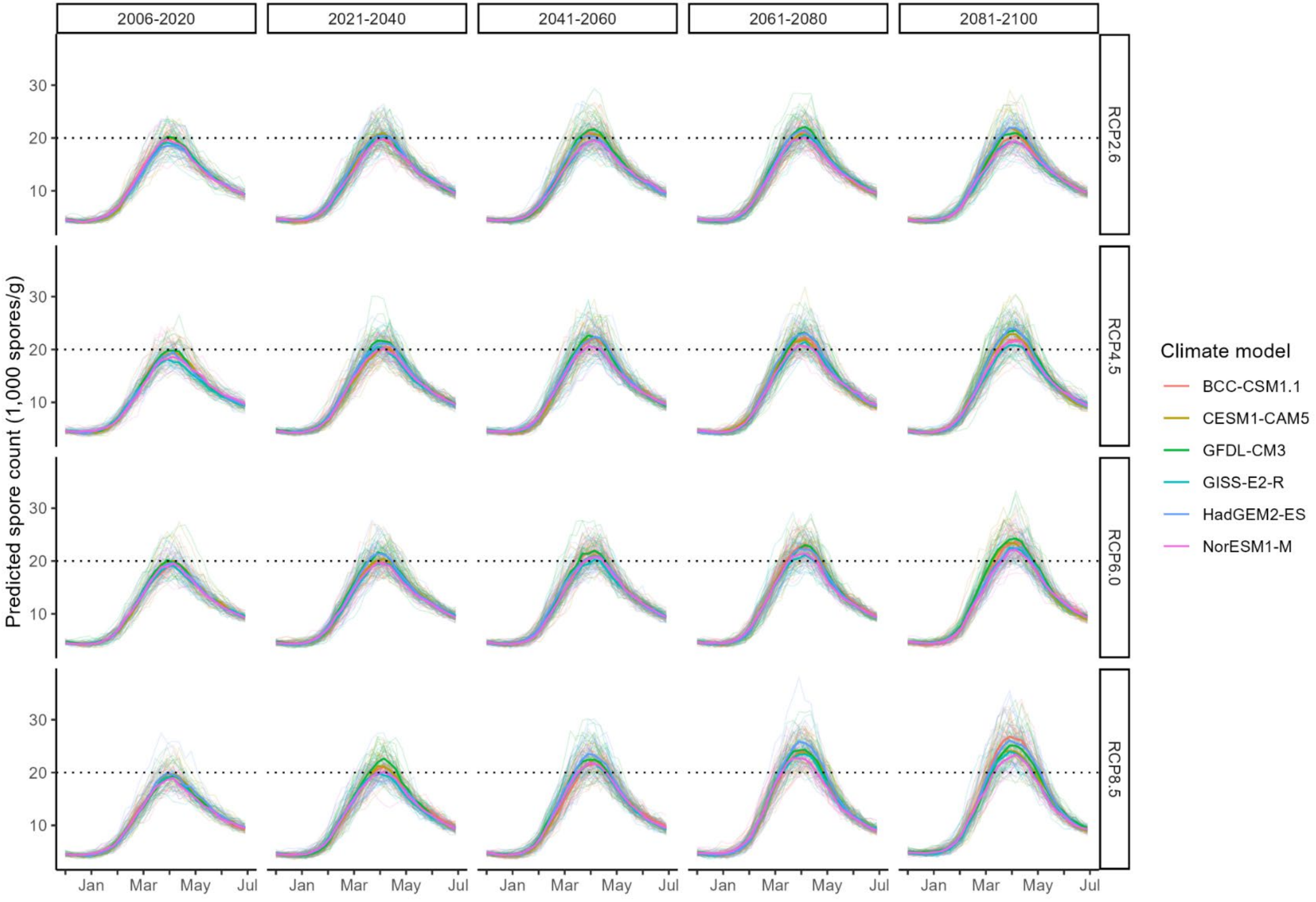

The content of this supplementary information has not been edited. All risk and liability rest with the authors.
